# Disparities in use modalities among adults who currently use cannabis, 2022-2023

**DOI:** 10.21203/rs.3.rs-5975991/v1

**Published:** 2025-04-02

**Authors:** Meman Diaby, Osayande Agbonlahor, Bethany Shorey Fennell, Joy L. Hart, Delvon T. Mattingly

## Abstract

**Purpose:** Following the legalization of cannabis in several U.S. states, the cannabis market has expanded, leading to a wider range of products including smoked, edible, and vape products which have variable health effects. This proliferation highlights the need for more research on patterns of current cannabis use among U.S. adults. **Methods:** We used combined data on adults who currently use (i.e., past 30-day use) cannabis (n=16,999) from the 2022 and 2023 National Survey on Drug Use and Health. We analyzed whether seven cannabis use modalities including smoking, vaping, dabbing, consuming edibles, taking pills, applying topicals, and absorbing sublingually/orally varied by age, sex, race and ethnicity, sexual orientation, education, income, geographic location, and state medical cannabis laws status by generating weighted proportion estimates and conducting multivariable logistic regression. Additionally, in a subanalysis, we examined differences in blunt use among U.S. adults who reported current cannabis use (n=12,355), employing similar methods to explore associations with demographic and socioeconomic factors. **Results:** Among adults who currently use cannabis, smoking was the most common cannabis use method (77.33%), followed by edibles (37.31%), vaping (34.75%), dabbing (15.01%), applying topicals (5.93%), absorbing sublingually/orally (4.53%), and taking pills (2.11%). Edibles were popular among adults aged 35-49 years (29.57%), whereas vaping was most common among young adults aged 18-25 years (29.80%). Females (vs. males) had lower odds of smoking cannabis (OR: 0.65; 95% CI: 0.57-0.75) and higher odds of applying topicals (OR: 2.92; 95% CI: 2.23-3.83). Non-Hispanic Black (vs. non-Hispanic White) respondents had higher odds of smoking cannabis (OR: 2.03; 95% CI: 1.51-2.74) and lower odds of consuming edibles (OR: 0.66; 95% CI: 0.56-0.77). Adults aged 50+ years (vs. 18-25) had greater odds of absorbing sublingually/orally (OR: 2.45; 95% CI: 1.59-3.76). In the subanalysis, we found that Non-Hispanic Black (vs. non-Hispanic White) adults had higher odds of blunt use (OR: 5.31; 95% CI: 4.23-6.65). **Conclusions:** Use modality disparities among adults who currently use cannabis highlight the need for tailored public health education and interventions, given the distinct health risks associated with each method of use.

### Introduction

Cannabis is one of the most commonly used substances among adults in the United States (U.S.) (Substance Abuse and Mental Health Services Administration, 2024). In 2023, 36.5% of young adults aged 18 to 25 years (approximately 12.4 million individuals) reported using cannabis in the past year, while 20.8% of adults aged 26 or older (about 45.5 million individuals) reported past-year use (Substance Abuse and Mental Health Services Administration, 2024). The legalization of cannabis use in numerous U.S. states and the increasing rates of current use among adults have resulted in public and scholarly debate about cannabis product regulation and associated health effects (Jeffers et al., 2024; Mattingly, Richardson, et al., 2024; *State-by-State Medical Marijuana Laws - ProCon.Org*, 2023). Although several studies suggest cannabis may be effective in treating certain types of pain, including pain associated with headaches, cancer, or chronic diseases, extensive research highlights various health-related risks depending on use modality (Solmi et al., 2023). Cannabis consumption, depending on the mode of use and its concentration of delta-9-tetrahydrocannabinol (THC), the primary psychoactive compound in cannabis, can impair cognitive functions such as memory and learning, potentially lead to dependency, exacerbate mental health conditions like schizophrenia, and contribute to respiratory issues with prolonged use (World Health Organization, 2016). Furthermore, cannabis use is associated with increased risk for several substance use disorders, with nearly 30% of cannabis users having a substance use disorder (D. S. Hasin et al., 2015).

Following the legalization of medical and recreational cannabis in many U.S. states, research on cannabis-derived products has increased, driven by concerns about the potential health effects of cannabis use and the rapid expansion of the cannabis industry (Hammond et al., 2022; D. Hasin & Walsh, 2021). Novel products designed to appeal to a wide range of consumers have been developed and marketed, particularly in states allowing the legal purchasing of cannabis (Borodovsky et al., 2016). These products, including edibles, drops, strips, lozenges, and sprays, vary in their cannabis composition, such as differences in THC or the inclusion of only cannabidiol (CBD), and in their potential health impacts (Goodman et al., 2020; Inman & Cservenka, 2024). Several studies have raised concerns about novel use methods (Spindle et al., 2019). Research on dabbing, for instance, suggests that the introduction of harmful components, such as solvents or pesticides during production, combined with the high concentrations of cannabinoids and THC, intensify psychoactive effects and increase risks of cannabis use disorder and cannabis poisoning (Alzghari et al., 2017; Inman & Cservenka, 2024).

Despite the growing availability of novel products, smoking and vaping remain the most popular cannabis use modalities (Inman & Cservenka, 2024; Lim et al., 2022; World Health Organization, 2016). Smoking cannabis has long been associated with respiratory complications, including bronchitis and increased mucus production, due to exposure to combustion byproducts (World Health Organization, 2016). The use of blunts, or cannabis rolled in cigar or tobacco leaves, also introduces additional risks related to tobacco exposure, including nicotine addiction, exacerbated cardiovascular and respiratory issues, and exposure to harmful toxins from tobacco leaves (Cooper & Haney, 2009; Sanchez et al., 2024). Additionally, smoking cannabis may increase cancer risk, though the evidence remains unclear and further research is needed to establish a definitive link (Ghasemiesfe et al., 2019; World Health Organization, 2016). Although vaping is often perceived as a safer alternative because it avoids combustion and tobacco smoke inhalation, research has shown that it may carry comparable or even greater risks to lung health, particularly when additives like vitamin E acetate are present (Correll & Vincent, 2024).

Over the years, the cannabis industry has developed a range of smokeless alternatives, often promoted for their convenience and potential to mitigate health risks associated with traditional smoking methods, yet these alternatives are not risk free (RTI International et al., 2016). Edibles, for instance, present unique risks of accidental overdose due to misuse (Huestis, 2007; Inman & Cservenka, 2024; Monte et al., 2019). Additionally, the average level of THC in all cannabis products has increased (Bero et al., 2023) and can vary substantially between products of the same type (e.g., edibles) increasing the risk of overconsumption (Inman & Cservenka, 2024). Other consumable products like drops, strips, lozenges, and sprays provide moderate durations of effect depending on their potency, but dosing misunderstandings can still lead to overconsumption (Huestis, 2007; Marquette et al., 2024).

This growing diversity in cannabis product options reflects not only industry innovation but also varying preferences and usage patterns across demographic groups (Inman & Cservenka, 2024; Leal & Moscrop-Blake, 2024; North et al., 2024; Schauer et al., 2016; Steigerwald et al., 2018). Males are more likely than females to engage in smoking, vaping, or dabbing, and sexual minorities are more likely than heterosexuals to engage in multimodal cannabis use (Leal & Moscrop-Blake, 2024; North et al., 2024). Conversely, females, older adults, and individuals with higher education tend to prefer less harmful cannabis forms, such as edibles or topical products (Leal & Moscrop-Blake, 2024; North et al., 2024). Black individuals are less likely than White individuals to engage in poly-modal use compared to single-modal use (Leal & Moscrop-Blake, 2024). These patterns have been observed in studies of young adults aged 22–30 years (North et al., 2024) and in nationally representative samples from the 2022 U.S. National Survey on Drug Use and Health (NSDUH) (Leal & Moscrop-Blake, 2024) as well as in other demographic studies (Inman & Cservenka, 2024; Mattingly et al., 2022; Schauer et al., 2016; Steigerwald et al., 2018). Additionally, residing in a state with medical marijuana laws is linked to greater use of edibles and other non-smoking cannabis products (Goodman et al., 2024; Shiplo et al., 2016).

Although research on cannabis use modalities has increased, much of this work has focused on youth and specific products such as vaping or smoking (D'Amico et al., 2020; Krauss et al., 2017; Peters et al., 2018; Wadsworth et al., 2022), and there is limited understanding of the use of other cannabis use modalities as well as the factors that influence modality choices, particularly among adult populations (Schauer et al., 2016; Subbaraman & Kerr, 2021). Additionally, more research is needed on novel cannabis products such as drops, lozenges, and pills (Wadsworth et al., 2022). This study aims to address gaps in the literature by examining disparities in cannabis use modalities among a national sample of U.S. adults who currently use cannabis (i.e., past 30-day use) by select sociodemographic characteristics and state medical cannabis laws status. We hypothesize that patterns of cannabis product use will vary across groups, including sex, race and ethnicity, sexual orientation, education, income, age, and states medical cannabis laws. We will observe disparities in use modalities (e.g., smoking vs. vaping) among adults who currently use cannabis.

### Methods

## Data and Participants

We used combined data from the 2022 and 2023 NSDUH, a study conducted by the Substance Abuse and Mental Health Services Administration (SAMHSA). The NSDUH is a repeated cross-sectional, nationally representative survey of the noninstitutionalized, civilian U.S. population aged 12 and above (*Substance Abuse and Mental Health Services Administration*, 2023). The NSDUH employs a multi-stage cluster sampling design to recruit U.S. youth and adults to examine substance use (i.e., tobacco, alcohol, and drugs), substance use disorders, mental health issues, as well as related treatment services. Further details regarding the NSDUH sampling methodology are available online (Center for Behavioral Health Statistics and Quality, 2023).

The total 2022 and 2023 NSDUH sample (n = 115,774) included both youth (aged 12–17 years) and adults (aged 18 + years). For our analysis, we restricted the sample to adults who reported using cannabis in any form in the past 30 days (n = 16,999).

We also conducted a subanalysis to incorporate blunt use as an additional use modality. Missingness patterns varied greatly between blunt use and the other cannabis use modalities, with 4,644 adults in the analytic sample missing data on blunt use. Missing values may be due in part to inconsistencies across sections of the survey. According to the 2023 NSDUH Public Use File Codebook, skipped responses in the section on blunt use were edited to align with prior responses; however, otherwise adjustments for consistency across sections were not generally made (Center for Behavioral Health Statistics and Quality, 2024). Thus, this subanalysis of disparities in blunt use included 12,355 respondents. A detailed depiction of our sample selection process is presented in Fig. 1.

### Measures

## Modalities of Cannabis Use

Participants were asked "During the past 30 days, in which of the following ways did you use marijuana or any cannabis product?" and could choose from the following responses: “smoking”, “vaping”, “dabbing, waxes, shatter, or concentrates”, “eating or drinking”, “putting drops, strips, lozenges, or sprays in your mouth or under your tongue”, “applying lotion, cream, or patches to your skin”, “taking pills”, or “some other way”. We derived seven dichotomous variables representing varying modalities of current cannabis use: (1) smoking cannabis, (2) vaping cannabis, (3) dabbing cannabis, (4) consuming edibles, (5) taking pills, (6) absorbing sublingually/orally, and (7) applying topicals.

Additionally, respondents were asked “How long has it been since you last smoked part or all of a cigar with marijuana in it?” and could choose a time frame. We derived the current blunt use variable from respondents who answered, “Within the past 30 days”.

### Sociodemographic Characteristics

We included the following sociodemographic characteristics: age (18–25, 26–34, 35–49, and 50 + years), sex (male and female), race and ethnicity (Hispanic, non-Hispanic (NH) White, NH Black, NH multiracial, and NH another race (e.g., American Indian/Alaska Native, Native Hawaiian/other Pacific Islander, or Asian)), sexual orientation (heterosexual, LGB + including participants who identified as gay, lesbian, or bisexual in both surveys, as well as those who selected "I use a different term," "I am not sure about my sexual identity," or "I do not know what this question is asking" in the 2023 NSDUH survey), educational attainment (less than high school (HS), HS graduate, some college, and college graduate or more), annual household income (less than $20,000, $20,000 to $49,999, $50,000 to $74,999, and $75,000+), and metropolitan status (large metro (a total population of 1 million or more), small metro (a total population of fewer than 1 million), and non-metro (rural)) based on the “Rural-Urban Continuum Codes” developed by the U.S. Department of Agriculture (Center for Behavioral Health Statistics and Quality, 2023).

### Substance use and mental health characteristics

We included a dichotomous variable for participants residing in states with medical cannabis laws (MCLs) at the time of the interview (yes/no) in the main analysis. Additionally, for the sensitivity analysis, we incorporated the following variables: current (i.e., past 30-day use) tobacco use (yes/no), current alcohol use (yes/no), current illicit drug use other than cannabis (i.e., cocaine, hallucinogens, heroin, inhalants, methamphetamine, psychotherapeutics) (yes/no), and current psychological distress (yes/no). Current psychological distress was defined as a score of 13 or higher on the six-item Kessler Psychological Distress Scale (K6) (Center for Behavioral Health Statistics and Quality, 2024).

## Statistical Analysis

We estimated the weighted proportion of cannabis use modalities, sociodemographic characteristics, and state MCLs status among adults who currently use cannabis. Then, we calculated the proportion of each sociodemographic characteristic and state MCLs by each cannabis use modality and compared differences in bivariate distributions using Chi-square tests of independence. We performed multivariable logistic regression models to estimate the adjusted associations between sociodemographic characteristics, state MCLs status, and cannabis use modalities. Additionally, we ran two sensitivity analyses and one supplementary analysis. The first sensitivity analysis fit multivariable logistic regression models to estimate the adjusted associations between sociodemographic characteristics, state MCLs status, additional covariates (including: current tobacco use, current alcohol use, current illicit drug use, and current psychological distress), and cannabis use modalities. The second sensitivity analysis fit multivariable logistic regression models to assess the adjusted associations between sociodemographic characteristics, state MCLs status, and cannabis use modalities among adults aged 18–25 who currently use cannabis. These sensitivity analyses were conducted to better understand patterns of cannabis use among younger adults, who have the highest prevalence of use, and to account for potential confounding by co-occurring substance use and mental health. As a supplementary analysis, we conducted a modified Poisson regression analysis to examine factors associated with the number of cannabis use modalities (range: 0–7) among adults who currently use cannabis.

We set the statistical significance level at 0.00625 based on the Bonferroni correction for multiple comparisons, given that we ran eight models for the main analysis, and reported the adjusted odds ratios with 95% confidence intervals (Armstrong, 2014). All analyses were conducted using STATA version 18.0, incorporating the *svy* command to account for the NSDUH study design, adjusting for non-response and selection probabilities (StataCorp, 2023).

### Results

## Sociodemographic Characteristics

Table 1 shows the proportion of participant characteristics among adults currently using cannabis. The age distribution included 21.14% aged 18–25 years, 24.27% aged 26–34 years, 27.03% aged 35–49 years, and 27.56% aged 50 + years. Regarding racial and ethnic composition, 64.53% of participants identified as NH White, 14.94% as Hispanic, 13.53% as NH Black, 3.61% as NH another race, and 3.38% as NH multiracial. The sample consisted of 43.21% female and 56.79% male participants. Most participants identified as heterosexual (79.68%), with 20.32% identifying as LGB+. Educational attainment among participants included 8.03% with less than a high school education, 27.78% who were high school graduates, 35.68% with some college education, and 28.51% with a college degree or higher. Annual household income varied, with 40.07% earning $75,000 or more, 27.72% earning $20,000-$49,000, 17.32% earning less than $20,000, and 14.88% earning $50,000-$74,999. Geographic distribution showed that 56.49% resided in large metropolitan areas, 31.49% in small metropolitan areas, and 12.02% in non-metropolitan areas. In terms of MCLs status, 79.75% of participants resided in a state with MCLs. Regarding cannabis use modalities, 77.33% reported smoking cannabis, 37.31% used edibles/drinks, 34.75% vaped, 15.01% dabbed, 5.93% applied topicals, 4.53% absorbed sublingually/orally, and 2.11% took pills.

Table 1
Prevalence of participant characteristics among adults who currently use cannabis (n = 16,999)

| | n (%) | 95% CI |
|---|---|---|
| **Sociodemographic characteristics, n (%)** | | |
| Age in years | | |
| 18–25 | 6768 (21.14) | 20.04, 22.29 |
| 26–34 | 4185 (24.27) | 23.26, 25.31 |
| 35–49 | 4245 (27.03) | 25.69, 28.41 |
| 50+ | 1801 (27.56) | 25.94, 29.24 |
| Sex | | |
| Male | 8460 (56.79) | 55.50, 58.07 |
| Female | 8539 (43.21) | 41.93, 44.50 |
| Race and ethnicity | | |
| Hispanic | 2837 (14.94) | 13.70, 16.28 |
| Non-Hispanic White | 10196 (64.53) | 62.98, 66.05 |
| Non-Hispanic Black | 2186 (13.53) | 12.52, 14.61 |
| Non-Hispanic multiracial | 1013 (3.38) | 2.96, 3.86 |
| Another non-Hispanic race [a] | 767 (3.61) | 3.11, 4.20 |
| Sexual orientation | | |
| Heterosexual | 12586 (79.68) | 78.43, 80.87 |
| LGB + [b] | 4413 (20.32) | 19.13, 21.57 |
| Educational attainment | | |
| Less than high school | 1886 (8.03) | 7.38, 8.74 |
| High school graduate | 4882 (27.78) | 26.06, 29.56 |
| Some college | 5658 (35.68) | 34.03, 37.35 |
| College graduate or more | 4573 (28.51) | 27.29, 29.77 |
| Annual household income | | |
| Less than $20,000 | 3612 (17.32) | 16.23, 18.48 |
| $20,000 to $49,999 | 5112 (27.72) | 26.29, 29.20 |
| $50,000 to $74,999 | 2398 (14.88) | 13.92, 15.89 |
| $75,000 or more | 5877 (40.07) | 38.50, 41.67 |
| Metropolitan status | | |
| Large metropolitan | 7713 (56.49) | 54.59, 58.36 |

| | n (%) | 95% CI |
|---|---|---|
| Small metropolitan | 6758 (31.49) | 29.72, 33.31 |
| Non-metropolitan | 2528 (12.02) | 10.67, 13.52 |
| **State with medical cannabis laws, n (%)** | | |
| No | 3037 (20.25) | 18.78, 21.81 |
| Yes | 13962 (79.75) | 78.19, 81.22 |
| **Cannabis use characteristics, n (%)** | | |
| Smoking cannabis (yes) | 13320 (77.33) | 76.08, 78.53 |
| Vaping cannabis (yes) | 6667 (34.75) | 35.55, 35.96 |
| Dabbing cannabis (yes) | 3247 (15.01) | 13.98, 16.11 |
| Consuming edibles (yes) | 6614 (37.31) | 35.95, 38.69 |
| Taking pills (yes) | 335 (2.11) | 1.73, 2.56 |
| Absorbing sublingually/orally [c] (yes) | 745 (4.53) | 3.96, 5.17 |
| Applying topicals (yes) | 1080 (5.93) | 5.25, 6.69 |

The counts (n) are unweighted and the percentages (%) are weighted

[a] Another non-Hispanic race includes respondents who identified as American Indian/Alaska Native, Native Hawaiian/other Pacific Islander, or Asian

[b] LGB + includes participants who identified as gay, lesbian, or bisexual in both surveys, as well as those who selected "I use a different term," "I am not sure about my sexual identity," or "I do not know what this question is asking" in the 2023 NSDUH survey

[c] Using drops/strips/lozenges/sprays

## Cannabis Use Modalities by Sociodemographic Characteristics, and state MCLs status

Table 2 displays the proportion of cannabis use by modality and sociodemographic characteristics and state MCLs status among adults who currently use cannabis. Compared to other age groups, adults aged 18–25 years had the highest proportion of dabbing (36.38%; $p < 0.001$) and vaping (29.80%; $p < 0.001$), those aged 35–49 had the highest proportion of consuming edibles (29.57%; $p < 0.001$) and taking cannabis pills (37.04%; $p > 0.05$), and those aged 50 + had the greatest proportion of smoking (26.55%; $p < 0.001$) absorbing sublingually/orally (38.21%; $p < 0.001$). Non-Hispanic White respondents had the highest proportion across all modalities, including smoking cannabis (62.12%; $p < 0.001$) and consuming edibles (70.40%; $p < 0.001$), while Non-Hispanic another race respondents reported the lowest proportion of vaping cannabis (3.14%; $p < 0.001$) and dabbing (3.64%; $p < 0.001$). Metropolitan status revealed that large metropolitan residents showed higher use proportion across all modalities (e.g., dabbing: 47.32%; $p < 0.001$), small metropolitan and non-metropolitan residents had their highest proportions in dabbing (37.75% and 14.93%; $p < 0.001$). For sexual orientation, participants who identified as heterosexual had higher proportion across all categories (e.g., vaping 74.19%; $p < 0.001$), and participants who identified as LGB + had their highest proportion in taking cannabis pills (28.62%; $p < 0.05$). For sex, male vs. female participants reported higher proportions of all cannabis use modalities except applying topicals where females had a higher proportion (68.49%; $p < 0.001$).

Educational attainment also revealed differences, with individuals having some college education showing the highest proportion of smoking (36.24%; $p < 0.001$), vaping (37.04%; $p < 0.05$), and applying topicals (46.35%; $p < 0.001$), whereas college graduates or more had the highest proportion of consuming edibles (40.53%; $p < 0.001$). Cannabis use also varied by income level, with individuals earning $75,000 or more having the highest proportion in most cannabis use modalities including smoking (35.53% $p < 0.001$) except dabbing, where those earning $20,000 to $49,999 (34.71%; $p < 0.001$) had the highest use proportion. Participants residing in states with MCLs had the highest proportion across all cannabis use modalities (e.g., taking pills 91.99%; $p < 0.001$) compared to those in states without MCLs.

Table 2
Prevalence of cannabis use by modalities, sociodemographic characteristics, and state medical cannabis laws status among adults who currently use cannabis (n = 16,999)

| | Cannabis use modalities, n (%) | | | | | | |
|---|---|---|---|---|---|---|---|
| Participant characteristics | Smoking cannabis (n = 13,320) | Vaping cannabis (n = 6,667) | Dabbing cannabis (n = 3,247) | Consuming edibles (n = 6,614) | Taking pills (n = 335) | Absorbing sublingually/orally [a] (n = 745) | Applying topicals (n = 1,080) |
| Age in years, n (%) | | | | | | | |
| 18–25 | 5644 (22.57)** | 3144 (29.80)** | 1825 (36.38)** | 2425 (20.90)** | 96 (13.26) | 192 (12.86)** | 300 (13.42)** |
| 26–34 | 3242 (24.37)** | 1661 (28.30)** | 764 (28.04)** | 1699 (25.93)** | 74 (19.26) | 175 (18.77)** | 258 (18.88)** |
| 35–49 | 3136 (26.51)** | 1484 (26.42)** | 567 (25.53)** | 1850 (29.57)** | 117 (37.04) | 257 (30.16)** | 347 (33.95)** |
| 50+ | 1298 (26.55)** | 378 (15.48)** | 91 (10.05)** | 640 (23.60)** | 48 (30.44) | 121 (38.21)** | 175 (33.74)** |
| Sex, n (%) | | | | | | | |
| Male | 6864 (59.16)** | 3397 (58.28) | 1802 (62.48)** | 3049 (51.40)** | 154 (52.47) | 322 (50.11)* | 334 (31.51)** |
| Female | 6456 (40.84)** | 3270 (41.72) | 1445 (37.52)** | 3565 (48.60)** | 181 (47.53) | 423 (49.89)* | 746 (68.49)** |
| Race and ethnicity, n (%) | | | | | | | |
| Hispanic | 2291 (15.11)** | 1170 (16.56)** | 628 (16.17)** | 942 (13.00)** | 46 (10.50) | 108 (12.45)** | 209 (15.75) |
| Non-Hispanic White | 7554 (62.12)** | 4358 (69.55)** | 1982 (68.11)** | 4443 (70.40)** | 228 (74.01) | 535 (76.73)** | 659 (66.21) |
| Non-Hispanic Black | 1997 (15.41)** | 458 (7.26)** | 218 (7.38)** | 581 (10.14)** | 28 (7.12) | 27 (3.56)** | 94 (10.27) |
| Non-Hispanic multiracial | 874 (3.78)** | 408 (3.48)** | 261 (4.70)** | 397 (3.22)** | 14 (5.16) | 38 (3.62)** | 75 (4.91) |
| Another non-Hispanic race [b] | 604 (3.58)** | 273 (3.14)** | 158 (3.64)** | 251 (3.24)** | 19 (3.22) | 37 (3.64)** | 43 (2.86) |
| Sexual orientation, n (%) | | | | | | | |
| Heterosexual | 9876 (79.76) | 4614 (74.19)** | 2239 (73.74)** | 4646 (74.83)** | 219 (71.38)* | 508 (75.08) | 728 (75.28)* |
| LGB + [c] | 3444 (20.24) | 2053 (25.81)** | 1008 (26.26)** | 1968 (25.17)** | 116 (28.62)* | 237 (24.92) | 352 (24.72)* |

| | Cannabis use modalities, n (%) | | | | | | |
|---|---|---|---|---|---|---|---|
| Educational attainment, n (%) | | | | | | | |
| Less than high school | 1688 (9.32)** | 682 (6.55)* | 540 (11.08)** | 443 (4.25)** | 37 (6.20)* | 56 (3.25)* | 103 (4.84)** |
| High school graduate | 4276 (31.15)** | 1924 (27.33)* | 1253 (37.45)** | 1432 (20.01)** | 60 (18.20)* | 154 (22.95)* | 290 (24.44)** |
| Some college | 4484 (36.24)** | 2357 (37.04)* | 1054 (35.85)** | 2245 (35.21)** | 110 (37.27)* | 257 (36.17)* | 435 (46.35)** |
| College graduate or more | 2872 (23.29)** | 1704 (29.09)* | 400 (15.61)** | 2494 (40.53)** | 128 (38.32)* | 278 (37.64)* | 252 (24.37)** |
| Annual household income, n (%) | | | | | | | |
| Less than $20,000 | 3166 (19.75)** | 1325 (15.23)* | 850 (21.70)** | 1121 (12.65)** | 58 (11.35) | 132 (11.58) | 232 (15.90) |
| $20,000 to $49,999 | 4271 (29.80)** | 1978 (26.60)* | 1159 (34.71)** | 1736 (23.90)** | 83 (25.69) | 200 (30.74) | 350 (28.42) |
| $50,000 to $74,999 | 1871 (14.93)** | 1000 (16.02)* | 440 (14.41)** | 926 (13.95)** | 47 (16.86) | 102 (15.18) | 172 (17.40) |
| $75,000 or more | 4012 (35.53)** | 2364 (42.16)* | 798 (29.17)** | 2831 (49.50)** | 147 (46.10) | 311 (42.49) | 326 (38.27) |
| Metropolitan status, n (%) | | | | | | | |
| Large metropolitan | 5934 (55.62)* | 2940 (56.29) | 1132 (47.32)** | 3051 (57.76) | 149 (57.01) | 305 (55.82) | 421 (48.63)* |
| Small metropolitan | 5312 (31.68)* | 2739 (31.85) | 1508 (37.75)** | 2648 (30.99) | 133 (32.25) | 324 (31.56) | 482 (37.55)* |
| Non-metropolitan | 2074 (12.70)* | 988 (11.86) | 607 (14.93)** | 915 (11.25) | 53 (10.75) | 116 (12.62) | 177 (13.83)* |
| State with medical cannabis laws | | | | | | | |
| No | 2396 (20.64) | 1231 (19.78) | 581 (19.83) | 1093 (19.33) | 30 (8.01)** | 95 (13.52)* | 154 (14.65)* |
| Yes | 10924 (79.36) | 5436 (80.22) | 2666 (80.17) | 5521 (80.67) | 305 (91.99)** | 650 (86.48)* | 926 (85.35)* |
| The counts (n) are unweighted and the percentages (%) are weighted | | | | | | | |
| *p < 0.05, **p < 0.001 from chi-square tests conducted within each group comparing distributions across sociodemographic characteristics, state medical cannabis laws status, and cannabis use modalities | | | | | | | |
| [a] Using drops/strips/lozenges/sprays | | | | | | | |

| Cannabis use modalities, n (%) |
|---|
| [b] Another non-Hispanic race includes respondents who identified as American Indian/Alaska Native, Native Hawaiian/other Pacific Islander, or Asian |
| [c] LGB + includes participants who identified as gay, lesbian, or bisexual in both surveys, as well as those who selected "I use a different term," "I am not sure about my sexual identity," or "I do not know what this question is asking" in the 2023 NSDUH survey. |

### Associations between Sociodemographic Characteristics, state MCLs status, and Cannabis Use Modalities

Table 3 presents the adjusted associations between sociodemographic characteristics, state MCLs status, and cannabis use modalities among adults currently using cannabis. Adults aged 26–34 years (vs. 18–25) had 31% lower odds of vaping (95% CI: 0.61–0.77), 28% lower odds of dabbing (95% CI: 0.61–0.85), but 44% higher odds of applying topicals (95% CI: 1.10–1.89). Those aged 35–49 years (vs. 18–25) had 49% lower odds of vaping (95% CI: 0.45–0.59), and 47% lower odds of dabbing (95% CI: 0.44–0.63), but higher odds of absorbing sublingually/orally (OR: 1.91; 95% CI: 1.35–2.72), and more than double the odds of taking pills (OR: 2.26; 95% CI: 1.34–3.81) and applying topicals (OR: 2.43; 95%CI: 1.82–3.25). Adults aged 50 + years (vs. 18–25) showed lower odds of smoking (OR: 0.74; 95% CI: 0.60–0.91), vaping (OR: 0.23; 95% CI: 0.19–0.30), dabbing (OR: 0.17; 95% CI: 0.11–0.25), and consuming edibles (OR: 0.71; 95% CI: 0.59–0.85), but higher odds of absorbing sublingually/orally (OR: 2.45; 95% CI: 1.59–3.76), and applying topicals (OR: 2.44; 95% CI: 1.67–3.56).

Table 3
Multivariable logistic regression estimating the association between sociodemographic characteristics, state medical cannabis laws status, and cannabis use modalities among adults who currently use cannabis (n = 16,999)

| | **Cannabis use modalities, AOR (95% CI) [a]** | | | | | | |
|---|---|---|---|---|---|---|---|
| **Participant characteristics** | Smoking cannabis | Vaping cannabis | Dabbing cannabis | Consuming edibles | Taking pills | Absorbing sublingually/orally [a] | Applying topicals |
| Age in years (ref: 18–25) | | | | | | | |
| 26–34 | 0.96 (0.83, 1.13) | **0.69 (0.61, 0.77)** | **0.72 (0.61, 0.85)** | 0.94 (0.79, 1.12) | 1.19 (0.62, 2.28) | 1.23 (0.83, 1.80) | **1.44 (1.10, 1.89)** |
| 35–49 | 0.86 (0.71, 1.05) | **0.51 (0.45, 0.59)** | **0.53 (0.44, 0.63)** | 1.02 (0.87, 1.19) | **2.26 (1.34, 3.81)** | **1.91 (1.35, 2.72)** | **2.43 (1.82, 3.25)** |
| 50+ | **0.74 (0.60, 0.91)** | **0.23 (0.19, 0.30)** | **0.17 (0.11, 0.25)** | **0.71 (0.59, 0.85)** | 1.88 (1.06, 3.35) | **2.45 (1.59, 3.76)** | **2.44 (1.67, 3.56)** |
| Sex (ref: male) | | | | | | | |
| Female | **0.65 (0.57, 0.75)** | **0.81 (0.72, 0.92)** | **0.68 (0.58, 0.80)** | **1.29 (1.13, 1.46)** | 1.04 (0.67, 1.63) | 1.20 (0.94, 1.53) | **2.92 (2.23, 3.83)** |
| Race and ethnicity (ref: non-Hispanic White) | | | | | | | |
| Hispanic | 1.03 (0.85, 1.24) | 0.83 (0.70, 0.98) | 0.74 (0.60, 0.93) | **0.73 (0.61, 0.86)** | 0.67 (0.41, 1.11) | 0.82 (0.54, 1.25) | 1.20 (0.89, 1.61) |
| Non-Hispanic Black | **2.03 (1.51, 2.74)** | **0.33 (0.27, 0.42)** | **0.36 (0.27, 0.49)** | **0.66 (0.56, 0.77)** | 0.55 (0.33, 0.93) | **0.25 (0.13, 0.47)** | 0.88 (0.59, 1.32) |
| Non-Hispanic multiracial | **1.90 (1.43, 2.53)** | 0.83 (0.65, 1.06) | 1.07 (0.82, 1.39) | 0.88 (0.66, 1.18) | 1.40 (0.41, 4.76) | 0.96 (0.53, 1.73) | 1.34 (0.80, 2.22) |
| Another non-Hispanic race [c] | 1.18 (0.75, 1.85) | 0.65 (0.47, 0.90) | 0.90 (0.56, 1.45) | 0.67 (0.46, 0.99) | 0.77 (0.27, 2.25) | 0.87 (0.44, 1.71) | 0.80 (0.36, 1.79) |
| Sexual orientation (ref: heterosexual) | | | | | | | |
| LGB + [d] | 0.95 (0.78, 1.16) | **1.37 (1.21, 1.56)** | **1.24 (1.07, 1.44)** | **1.51 (1.31, 1.74)** | **1.94 (1.25, 3.01)** | 1.60 (1.09, 2.33) | 1.31 (0.98, 1.75) |
| Educational attainment (ref: less than high school) | | | | | | | |

| | Cannabis use modalities, AOR (95% CI) [a] | | | | | | |
|---|---|---|---|---|---|---|---|
| High school graduate | 0.87 (0.63, 1.19) | **1.37 (1.14, 1.65)** | 1.07 (0.85, 1.36) | **1.42 (1.16, 1.73)** | 0.77 (0.37, 1.61) | 1.91 (1.17, 3.11) | 1.40 (0.85, 2.31) |
| Some college | **0.56 (0.42, 0.74)** | **1.44 (1.20, 1.73)** | 0.78 (0.63, 0.96) | **2.06 (1.63, 2.60)** | 1.16 (0.61, 2.24) | **2.27 (1.41, 3.65)** | 1.84 (1.15, 2.94) |
| College graduate or more | **0.31 (0.24, 0.40)** | 1.32 (1.06, 1.64) | **0.43 (0.34, 0.54)** | **3.55 (2.73, 4.62)** | 1.37 (0.72, 2.60) | **2.94 (1.85, 4.65)** | 1.19 (0.69, 2.04) |
| Annual household income (ref: less than $20,000) | | | | | | | |
| $20,000 to $49,999 | 0.73 (0.57, 0.93) | 1.11 (0.95, 1.31) | 1.04 (0.86, 1.27) | 1.19 (1.00, 1.42) | 1.33 (0.76, 2.34) | 1.53 (1.07, 2.18) | 1.11 (0.79, 1.5) |
| $50,000 to $74,999 | **0.58 (0.43, 0.76)** | **1.28 (1.08, 1.52)** | 0.78 (0.61, 1.01) | 1.19 (0.97, 1.47) | 1.56 (0.60, 4.04) | 1.30 (0.79, 2.14) | 1.27 (0.85, 1.88) |
| $75,000 or more | **0.44 (0.34, 0.57)** | **1.32 (1.09, 1.59)** | **0.70 (0.57, 0.86)** | **1.72 (1.42, 2.08)** | 1.37 (0.99, 1.90) | 1.14 (0.76, 1.73) | 1.13 (0.77, 1.66) |
| Metropolitan status (ref: large metropolitan) | | | | | | | |
| Small metropolitan | 0.94 (0.80, 1.11) | 0.98 (0.86, 1.12) | 1.27 (1.05, 1.54) | 1.07 (0.93, 1.22) | 1.06 (0.73, 1.55) | 0.99 (0.76, 1.30) | 1.38 (1.05, 1.81) |
| Non-metropolitan | 1.11 (0.86, 1.44) | 1.02 (0.84, 1.25) | 1.33 (1.02, 1.73) | 1.12 (0.94, 1.33) | 1.00 (0.58, 1.71) | 1.04 (0.69, 1.57) | 1.34 (0.93, 1.92) |
| State with medical cannabis laws (ref: No) | | | | | | | |
| Yes | 0.98 (0.82, 1.17) | 1.06 (0.92, 1.23) | 1.13 (0.92, 1.38) | 1.09 (0.92, 1.28) | **2.91 (1.54, 5.47)** | 1.54 (1.00, 2.39) | **1.52 (1.17, 1.96)** |
| Bolded adjusted odds ratios and 95% confidence intervals indicate statistical significance (p < 0.00625) | | | | | | | |
| AORs and 95% CIs not bolded despite appearing statistically significant did not remain significant after Bonferroni correction | | | | | | | |
| [a] Odds ratios are adjusted for age, sex, race and ethnicity, sexual orientation, educational attainment, annual household income, metropolitan status, and state medical cannabis laws status | | | | | | | |
| [b] Using drops/strips/lozenges/sprays | | | | | | | |

| Cannabis use modalities, AOR (95% CI) [a] |
|---|
| [c] Another non-Hispanic race includes respondents who identified as American Indian/Alaska Native, Native Hawaiian/other Pacific Islander, or Asian |
| [d] LGB + includes participants who identified as gay, lesbian, or bisexual in both surveys, as well as those who selected "I use a different term," "I am not sure about my sexual identity," or "I do not know what this question is asking" in the 2023 NSDUH survey |

Female (vs. male) participants had 35% lower odds of smoking (95% CI: 0.57–0.75), 19% lower odds of vaping (95% CI: 0.72–0.92), and 32% lower odds of dabbing (95% CI: 0.58–0.80), but higher odds of consuming edibles (OR: 1.29; 95%CI: 1.13–1.46), and more than double the odds of applying topicals (OR: 2.92; 95% CI: 2.23–3.83).

Hispanic (vs. NH White) participants had 27% lower odds of consuming edibles (95% CI: 0.61–0.86). NH Black adults (vs. NH White) had lower odds of vaping (OR: 0.33; 95% CI: 0.27–0.42), dabbing (OR: 0.36; 95% CI: 0.27–0.49), consuming edibles (OR: 0.66; 95% CI: 0.56–0.77), and absorbing sublingually/orally (OR: 0.25; 95% CI: 0.13–0.47), but higher odds of smoking (OR: 2.03; 95% CI: 1.51–2.74).

LGB+ (vs. heterosexual) participants had greater odds of vaping (OR: 1.37; 95% CI: 1.21–1.56), dabbing (OR: 1.24; 95% CI: 1.07–1.44), consuming edibles (OR: 1.51; 95% CI: 1.31–1.74), and taking pills (OR: 1.94; 95% CI: 1.25–3.01).

High school graduates (vs. less than high school) had greater odds of vaping (OR: 1.37; 95% CI: 1.14–1.65), and consuming edibles (OR: 1.42; 95% CI: 1.16–1.73). Participants with some college education (vs. less than high school) had greater odds of vaping (OR: 1.44; 95% CI: 1.20–1.73), consuming edibles (OR: 2.06; 95% CI: 1.63–2.60), and sublingually/orally absorption (OR: 2.27; 95% CI: 1.41–3.65), but lower odds of smoking (OR: 0.56; 95% CI: 0.42–0.74). College graduates (vs. less than high school) had lower odds of smoking (OR: 0.31; 95% CI: 0.24–0.40), and dabbing (OR: 0.43; 95% CI: 0.34–0.54), but higher odds of absorbing sublingually/orally (OR: 2.94; 95% CI: 1.85–4.65), and threefold higher odds of consuming edibles (OR: 3.55; 95% CI: 2.73–4.62).

Participants earning $50,000 to $74,999 (vs. less than $20,000) had 42% lower odds of smoking (95% CI: 0.43–0.76), but 28% higher odds of vaping (95% CI: 1.08–1.52). Individuals earning $75,000 or more (vs. less than $20,000) had 56% lower odds of smoking (95% CI: 0.34–0.57), and 30% lower odds of dabbing (95% CI: 0.57–0.86), but higher odds of vaping (OR: 1.32; 95% CI: 1.09–1.59) and consuming edibles (OR: 1.72; 95% CI: 1.42–2.08).

Participants residing in a state with MCLs (vs. not residing in state with MCLs) had higher odds of taking pills (OR: 2.91; 95%CI: 1.54–5.47) and applying topicals (OR: 1.52; 95% CI: 1.17–1.96).

## Proportion and adjusted associations of blunt use by sociodemographic characteristics, and state MCLs status

Table 4 presents the weighted prevalence and adjusted associations of blunt use by sociodemographic characteristics and state MCLs status among current cannabis users. Compared to other age groups, people aged 18–25 years had a higher proportion of blunt use (31.04%, $p < 0.001$). Similarly, participants who reported being male (57.71%, $p > 0.05$), NH White (47.48%, $p < 0.001$), heterosexual (76.93%, $p < 0.05$), and high school graduates (38.52%, $p < 0.001$) as well as those earning $20,000-$49,000 (34.50%, $p < 0.001$), living in a large metropolitan area (54.47%, $p > 0.05$), and residing in a state with MCLs (74.78%, $p < 0.001$) had higher proportions of blunt use compared to their counterparts within each respective category.

Table 4
Proportions and adjusted associations of blunt use by sociodemographic characteristics, and state medical cannabis laws status among current cannabis users (n = 12,355)

| | **n (%)** | |
|---|---|---|
| Participant characteristics | Blunting (n = 4,944) | AOR (95% CI) [a] |
| Age in years | | |
| 18–25 | 2488 (31.04)** | REF |
| 26–34 | 1241 (29.59)** | 0.89 (0.73, 1.08) |
| 35–49 | 1052 (29.45)** | 0.80 (0.68, 0.95) |
| 50+ | 163 (9.91)** | **0.29 (0.22, 0.39)** |
| Sex, n (%) | | |
| Male | 2530 (57.71) | REF |
| Female | 2414 (42.29) | 1.12 (0.97, 1.30) |
| Race and ethnicity | | |
| Hispanic | 948 (17.01)** | 1.41 (1.10, 1.80) |
| Non-Hispanic White | 2164 (46.48)** | REF |
| Non-Hispanic Black | 1262 (28.94)** | **5.31 (4.23, 6.65)** |
| Non-Hispanic multiracial | 377 (4.52)** | **1.69 (1.17, 2.43)** |
| Another non-Hispanic race [b] | 193 (3.05)** | 1.58 (1.08, 2.32) |
| Sexual orientation | | |
| Heterosexual | 3634 (76.93) | REF |
| LGB + [c] | 1310 (23.07) | 0.90 (0.77, 1.04) |
| Educational attainment | | |
| Less than high school | 825 (12.39)** | REF |
| High school graduate | 1950 (38.52)** | 0.99 (0.79, 1.25) |
| Some college | 1661 (37.02)** | **0.65 (0.52, 0.81)** |
| College graduate or more | 508 (12.06)** | **0.25 (0.19, 0.33)** |
| Annual household income | | |
| Less than $20,000 | 1377 (24.03)** | REF |
| $20,000 to $49,999 | 1832 (34.50)** | 0.86 (0.73, 1.01) |
| $50,000 to $74,999 | 666 (15.78)** | 0.75 (0.59, 0.97) |
| $75,000 or more | 1069 (25.68)** | **0.60 (0.49, 0.75)** |
| Metropolitan status | | |

| | n (%) | |
|---|---|---|
| Large metropolitan | 2250 (54.47) | REF |
| Small metropolitan | 1998 (32.97) | 1.03 (0.88, 1.20) |
| Non-metropolitan | 696 (12.56) | 1.23 (0.96, 1.56) |
| State with medical cannabis laws | | |
| No | 1058 (25.22)** | REF |
| Yes | 3886 (74.78)** | 0.84 (0.71, 0.98) |
| The counts (n) are unweighted and the percentages (%) are weighted | | |
| *p < 0.05, ** p < 0.001 from chi-square tests conducted within each group comparing distributions across sociodemographic characteristics, state medical cannabis laws status, and cannabis use modalities | | |
| Bolded adjusted odds ratios and 95% confidence intervals indicate statistical significance (p < 0.00625) | | |
| AORs and 95% CIs not bolded despite appearing statistically significant did not remain significant after Bonferroni correction | | |
| [a] Odds ratios are adjusted for age, sex, race and ethnicity, sexual orientation, educational attainment, annual household income, metropolitan status, and state medical cannabis laws status | | |
| [b] Another non-Hispanic race includes respondents who identified as American Indian/Alaska Native, Native Hawaiian/other Pacific Islander, or Asian | | |
| [c] LGB + includes participants who identified as gay, lesbian, or bisexual in both surveys, as well as those who selected "I use a different term," "I am not sure about my sexual identity," or "I do not know what this question is asking" in the 2023 NSDUH survey. | | |

For the adjusted associations of blunt use by sociodemographic characteristics and state MCLs status among current cannabis users, we observed that NH Black (vs. NH White) adults had five times (OR: 5.31; 95% CI: 4.23–6.65) higher odds of current blunt use, and NH multiracial (vs. NH White) adults had higher odds (OR: 1.69; 95% CI: 1.17–2.43) of current blunt use. Participants aged 50 + (vs. 18–25) years had 71% (95% CI: 0.22–0.39) lower odds of blunt use. Additionally, participants with some college education (vs. less than high school) and college graduates (vs. less than high school) had respectively 35% (95% CI: 0.52–0.81) and 75% (95% CI: 0.19–0.33) lower odds of blunt use. Furthermore, participants earning $75,000 or more had 40% (95% CI: 0.49–0.75) lower odds of blunt use.

## Sensitivity analysis

Tables S1 and S2 present the adjusted associations between sociodemographic, mental health, and substance use characteristics, and cannabis use modalities among adults who currently use cannabis. After adjusting for the additional covariates, we observed some changes in statistical significance. For example, we found that high school graduates (vs. less than high school) had significantly higher odds of absorbing sublingually/orally (OR: 2.00; 95% CI: 1.23–3.25); participants aged 35–49 (vs. 18–25) no longer had significantly higher odds of taking pills; and individuals earning $75,000 or more (vs. less than $20,000) were no longer significantly lower odds to engage in dabbing.

Tables S4 and S5 present the adjusted associations between sociodemographic characteristics, state MCLs status, and cannabis use modalities among adults aged 18–25 who currently use cannabis. After restricting the sample to this age group, we observed some changes in statistical significance. For example, LGB + individuals (vs.

heterosexual) no longer had significantly higher odds of dabbing or taking pills, and females (vs. males) no longer had significantly lower odds of consuming edibles.

## Supplementary Analysis

Table S3 presents the adjusted associations between sociodemographic characteristics, state MCLs status, and the number of cannabis use modalities among adults who currently use cannabis. Participants aged 50 and older (vs. 18–25) had 26% lower prevalence of using an additional cannabis modality (95% CI: 0.70–0.77). NH Black participants (vs. NH White) had 19% lower prevalence of using an additional cannabis modality (95% CI: 0.77–0.85), whereas LGB+ (vs. heterosexual) participants had 13% higher prevalence of using an additional cannabis modality (CI: 1.09–1.17). Participants having some college education (vs. less than high school) exhibited 9% higher prevalence of using an additional cannabis modality (95% CI: 1.05–1.14). Additionally, residing in a state with MCLs (vs. not residing in state with MCLs) was associated with a 5% higher prevalence of using an additional cannabis modality (95% CI: 1.02–1.09).

## Discussion

This study examined differences in cannabis use modalities among U.S. adults who currently use cannabis, revealing notable differences across sociodemographic groups, including variations in modality preferences by age, sex, race and ethnicity, sexual orientation, educational attainment, annual household income, and state MCLs status. We observed that females, compared to males, had higher odds of consuming edibles; NH Black respondents, compared to NH White respondents, had higher odds of smoking cannabis; adults aged 50 years or older, compared to those aged 18–25 years, had greater odds of applying topicals; LGB+, compared to heterosexual, participants had higher odds of dabbing; NH Black adults, compared to NH White adults, had higher odds of using blunts; and participants residing in states with MCLs, compared to participants residing in states without MCLs, had higher odds of taking cannabis pills.

Consistent with previous research, our findings indicate that smoking remains the most common cannabis use modality across all groups (Inman & Cservenka, 2024; Schauer et al., 2016; Singh et al., 2016). This finding, especially in light of past research, raises significant public health concerns, as numerous studies have demonstrated that smoking cannabis is associated with various health risks, including respiratory issues, cardiovascular problems, and a higher potential for addiction (Inman & Cservenka, 2024). Smoking cannabis also increases the likelihood of co-use with tobacco, which can exacerbate health problems as well as open the possibility of tobacco-related ones, including cancer and lung disease (Cohn & Chen, 2022; Inman & Cservenka, 2024; Reboussin et al., 2021). These findings support the call for enhanced public health campaigns to raise awareness about the risks associated with smoking cannabis, while also emphasizing harm reduction strategies such as promoting safer consumption methods, educating users about dosing and potency, and providing resources for cessation or reduced use, especially considering the widespread prevalence (Murphy et al., 2015).

Although previous research has often ranked vaping as the second most common mode of cannabis consumption (Baldassarri et al., 2020; Cuttler et al., 2016; Wadsworth et al., 2022; Watson et al., 2022), our findings suggest a more nuanced picture for adults. Depending on the sociodemographic group, either vaping or consuming edibles emerged as the second most common method of cannabis use, aligning with findings from a study using the 2016 Behavioral Risk Factor Surveillance System (Schauer et al., 2020). Broader access to commercial cannabis products, shifting health perceptions, and regulatory changes may be driving this trend (Florimbio et al., 2023; National Academies of Sciences et al., 2024). This idea aligns with recent studies suggesting that edibles are increasing in popularity, particularly in states where cannabis has been legalized (Leal & Moscrop-Blake, 2024; Reboussin et al., 2019; Schauer et al., 2016). This shift in preference should be closely monitored, as both vaping and edible consumption pose health risks (Russell et al., 2018).

Among the risks of edibles are the delayed onset of effects and the difficulties in dosage control, which can lead to overconsumption and severe intoxication among adult users (Allen et al., 2017; Lamy et al., 2016; Reboussin et al., 2019). Also, inadequate labeling and unclear packaging of cannabis products contribute to significant consumer safety concerns (RTI International et al., 2016). These concerns highlight the need for stricter regulation and clearer labeling of cannabis products (Hancock-Allen et al., 2015; MacCoun & Mello, 2015; Onders et al., 2016). It is crucial to educate consumers on the risks associated with this cannabis consumption method and ensure that product labels provide accurate information about potency and recommended dosages (Allen et al., 2017; Lamy et al., 2016; Reboussin et al., 2019; Vandrey et al., 2015).

Our study identified significant variation in cannabis use across sociodemographic groups, consistent with findings from previous research (Cuttler et al., 2016; Friese et al., 2016; Gallup Inc., 2024; Jeffers et al., 2021; Leal & Moscrop-Blake, 2024; Mattingly et al., 2022; Schauer et al., 2020). The finding that NH Black individuals had higher odds of smoking cannabis underscores the critical need for targeted interventions to address this disparity. Potential underlying causes may include systemic inequities such as disproportionate exposure to chronic stress, limited access to cessation resources, and cannabis industry targeting of Black communities (Matsuzaka & Knapp, 2020; Mattingly et al., 2020; Mattingly, Mezuk, et al., 2024; Unger et al., 2020). Public health initiatives should prioritize culturally tailored cessation programs and reduction strategies, developed collaboratively with trusted community organizations, to ensure community engagement and efficacy (Montgomery et al., 2020). These efforts should focus on reducing smoking prevalence while addressing broader structural determinants that perpetuate disparities.

In line with these disparities and consistent with previous research (Montgomery et al., 2022; Sanchez et al., 2024; Schauer et al., 2017), we found that NH Black individuals had significantly higher odds of blunt use than NH White individuals. Prior literature highlights several potential causes for this disparity, including targeted advertising of tobacco and cannabis products in NH Black communities, socioeconomic inequities that limit access to healthcare and cessation programs, cultural acceptance of blunt use as a social practice, and coping mechanisms for managing systemic stress and discrimination (Montgomery et al., 2020, 2022; Sanchez et al., 2024). To address this disparity and reduce the prevalence of cannabis blunt use among NH Black individuals, previous studies have emphasized several key actions, including enhancing public education on health effects, regulating tobacco industry practices such as flavored wrappers and single cigarillo sales, providing tailored mental health and clinical support for blunt users, increasing funding for research, and improving survey tools for accurate data collection (Montgomery et al., 2019, 2022; Sanchez et al., 2024).

Additionally, our findings reveal significant sociodemographic differences in high-risk cannabis use modalities, with younger adults, males, and individuals with lower incomes more likely to engage in practices such as dabbing and smoking. Higher odds of dabbing among LGB + individuals emphasize the need for tailored interventions addressing the intersection of sexual minority status and cannabis use (Dyar, 2022; Romm et al., 2023). Public health campaigns may benefit from offering accessible, gender-specific, and age-appropriate prevention and cessation resources. Addressing economic barriers, such as through subsidized programs, can further support low-income populations (Jeffers et al., 2021). Also, the link between lower odds of dabbing and higher education and income underscores the importance of addressing structural factors shaping cannabis use behaviors. Targeted harm reduction strategies and regulatory measures, like potency limits, clear labeling, and marketing and advertising restrictions, are essential to mitigate risks for vulnerable groups.

Furthermore, our supplementary findings reveal significant sociodemographic and states medical cannabis laws status differences in multimodal cannabis use, aligning with previous studies (North et al., 2024; Schauer et al., 2020). Lower prevalence of using an additional cannabis modality among older adults and non-Hispanic Black participants suggest disparities in access or cultural norms, consistent with prior research on cannabis consumption patterns (North et al., 2024; Schauer et al., 2020). Conversely, higher prevalence among LGB + individuals and those with some college education may reflect greater exposure or differing motivations, aligning with prior studies that have found higher odds of multimodal cannabis use in these demographic groups compared to their counterparts (North et al., 2024). The association between medical cannabis laws and increased multimodal use reinforces earlier findings linking legalization to broader product availability and acceptance (Schauer et al., 2020). These results provide additional context for understanding cannabis use disparities, and given that multimodal use may increase THC exposure and associated health risks compared to exclusive single-method use, further research is needed to assess its long-term implications (Swan et al., 2021).

Adult cannabis consumption has been increasing steadily, particularly with the legalization and wider availability of cannabis products (Compton et al., 2016; D. Hasin & Walsh, 2021; Mattingly, Richardson, et al., 2024; Palamar et al., 2021), and this study's findings shed additional light on use modalities. Understanding the patterns of cannabis use among adults, especially with respect to less-studied products, is crucial for informing public health policies, enhancing clinical guidelines, and improving consumer safety. Given the increasing prevalence of cannabis use among U.S. adults, understanding use disparities can help in tailoring educational and harm-reduction efforts more effectively, particularly for populations that may be at greater risk of harm. Future research may benefit from exploring the intersectionality of sociodemographic factors, as examining how multiple factors—such as race, gender, income, and education—intersect could provide deeper insights into disparities in cannabis use and groups more at-risk for associated health outcomes (Mereish & Bradford, 2014; Schuler et al., 2020).

## Limitations

This study has several limitations. First, reliance on self-reported responses may introduce recall and social desirability bias. Second, the NSDUH excludes certain populations, such as those in institutional settings or without stable housing, limiting the generalizability of findings to those population groups. Third, data on blunt use was not available for about 27% of current cannabis users, and their exclusion may have introduced selection bias for the subanalysis. Additionally, this study does not account for the frequency or intensity of cannabis use, nor the potency of cannabis products, which may vary across use modalities and influence health outcomes. Finally, unmeasured factors, such as additional state-level policy differences (e.g., recreational cannabis laws), not addressed in this analysis could impact cannabis use patterns by geography. Despite these limitations, this study provides valuable insights into cannabis use disparities by use modality among adults who currently use cannabis.

### Conclusions

Our study identifies notable differences in cannabis use modalities across sociodemographic groups, particularly for smoking, vaping, and consuming edibles. These findings point to the need for targeted public health interventions to address the specific risks associated with each method of consumption. Given the distinct harms linked to various cannabis administration modes, efforts should focus on educating consumers, improving product labeling, and promoting cannabis cessation. Additionally, further research is crucial to understanding emerging trends and the impact of less common cannabis products, such as drops and lozenges. Insights from such work can inform more effective policies and public health strategies to mitigate the risks of cannabis use in a rapidly evolving landscape.

## Abbreviations

U.S.
United States
THC
Tetrahydrocannabinol
AOR
Adjusted odds ratio
vs
Versus
MCLs
Medical cannabis laws
CI
Confidence interval
OR
Odds ratio
*P*
P-value
NSDUH
National Survey on Drug Use and Health
NH
Non-Hispanic
HS
High school

## Declarations

Not applicable

**Ethics approval and consent to participate:**

This study was deemed exempt from review by the University of Kentucky institutional review board given use of publicly available, deidentified data, and followed STROBE reporting guidelines (von Elm et al., 2007).

**Consent for publication:**

All authors reviewed and approved the final version of the manuscript for submission.

**Availability of data and materials:**

The data that support the findings of this study are openly available at the Substance Abuse and Mental Health Services Administration (SAMHSA) (https://www.samhsa.gov/data/data-we-collect/nsduh-national-survey-drug-use-and-health)

**Competing interests:**

The authors declare no competing interests.

**Funding:**

This research was supported, in part, by the National Institute on Drug Abuse of the National Institutes of Health (NIH) under Award Number R25DA054015 (MPIs: Obasi and Reitzel). Dr. Mattingly's time was also supported by the National Cancer Institute of the NIH under Award Number 3R01CA251478-04S1 (PI: Rose).

**Authors' contributions:**

MD performed the subanalysis, sensitivity analyses, and supplementary analysis; verified and revised the original analysis; drafted the initial manuscript; and finalized the manuscript. JLH, OA, and BSF contributed to conceptualization, data interpretation, and critically reviewed and edited the manuscript. DTM designed and conducted the original analysis, supervised the study, and critically reviewed and edited the manuscript. All authors read and approved the final version of the manuscript.

**Acknowledgements:**

Not applicable

**Authors' information:**

Not applicable

## Figures

**Figure 1. Study flowchart describing the selection of the analytic samples, the 2022 and 2023 National Survey on Drug Use and Health (NSDUH).**

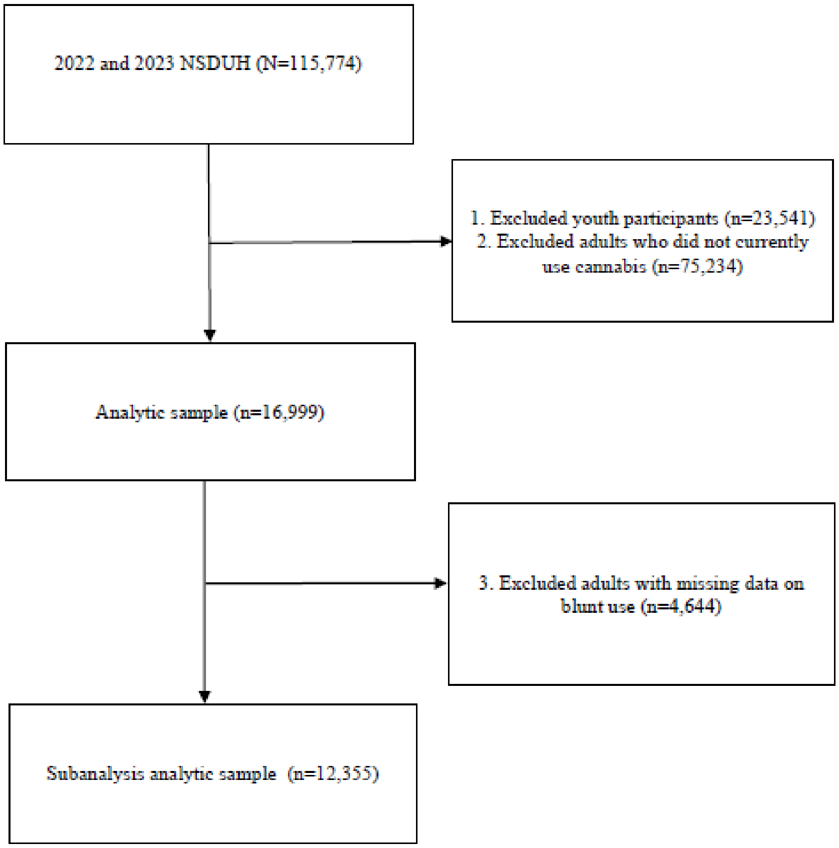

#### Figure 1

See image above for figure legend

### Supplementary Files

This is a list of supplementary files associated with this preprint. Click to download.

- SupplementarytablesS1S5Diabyetal.pdf
